# Anaesthetic Impairment of Immune Function Is Mediated via GABA_A_ Receptors

**DOI:** 10.1371/journal.pone.0017152

**Published:** 2011-02-24

**Authors:** Daniel W. Wheeler, Andrew J. Thompson, Federico Corletto, Jill Reckless, Justin C. T. Loke, Nicolas Lapaque, Andrew J. Grant, Pietro Mastroeni, David J. Grainger, Claire L. Padgett, John A. O'Brien, Nigel G. A. Miller, John Trowsdale, Sarah C. R. Lummis, David K. Menon, John S. Beech

**Affiliations:** 1 Division of Anaesthesia, University of Cambridge, Addenbrooke's Hospital, Cambridge, United Kingdom; 2 Department of Biochemistry, University of Cambridge, Cambridge, United Kingdom; 3 Department of Medicine, University of Cambridge, Addenbrooke's Hospital, Cambridge, United Kingdom; 4 Department of Pathology, University of Cambridge, Cambridge, United Kingdom; 5 Department of Veterinary Medicine, University of Cambridge, Cambridge, United Kingdom; 6 Neurobiology Division, MRC Laboratory of Molecular Biology, Cambridge, United Kingdom; City of Hope, United States of America

## Abstract

**Background:**

GABA_A_ receptors are members of the Cys-loop family of neurotransmitter receptors, proteins which are responsible for fast synaptic transmission, and are the site of action of wide range of drugs [1]. Recent work has shown that Cys-loop receptors are present on immune cells, but their physiological roles and the effects of drugs that modify their function in the innate immune system are currently unclear [2]. We are interested in how and why anaesthetics increase infections in intensive care patients; a serious problem as more than 50% of patients with severe sepsis will die [3]–[6]. As many anaesthetics act via GABA_A_ receptors [7], the aim of this study was to determine if these receptors are present on immune cells, and could play a role in immunocompromising patients.

**Principal Findings:**

We demonstrate, using RT-PCR, that monocytes express GABA_A_ receptors constructed of α1, α4, β2, γ1 and/or δ subunits. Whole cell patch clamp electrophysiological studies show that GABA can activate these receptors, resulting in the opening of a chloride-selective channel; activation is inhibited by the GABA_A_ receptor antagonists bicuculline and picrotoxin, but not enhanced by the positive modulator diazepam. The anaesthetic drugs propofol and thiopental, which can act via GABA_A_ receptors, impaired monocyte function in classic immunological chemotaxis and phagocytosis assays, an effect reversed by bicuculline and picrotoxin.

**Significance:**

Our results show that functional GABA_A_ receptors are present on monocytes with properties similar to CNS GABA_A_ receptors. The functional data provide a possible explanation as to why chronic propofol and thiopental administration can increase the risk of infection in critically ill patients: their action on GABA_A_ receptors inhibits normal monocyte behaviour. The data also suggest a potential solution: monocyte GABA_A_ receptors are insensitive to diazepam, thus the use of benzodiazepines as an alternative anesthetising agent may be advantageous where infection is a life threatening problem.

## Introduction

The anaesthetic drug, propofol, is a first line agent for sedation of critically ill patients on intensive care, facilitating potentially life-saving invasive treatments such as mechanical ventilation [1]. Its use, however, can have serious side effects, such as an increase in the incidence of secondary pneumonia from 35% to 53% [2]. Thiopental is another intravenous anaesthetic used in intensive care patients, usually for the management of patients with refractory status epilepticus or intracranial hypertension [1], but again can impair immune function, for example by suppressing bone marrow haematopoiesis [3]. The extent to which these drugs immunocompromise patients, and the contribution of this immunological impairment to the development of septicaemia on the intensive care unit has not been studied. However, a large proportion of critically ill patients have a primary diagnosis of septicaemia or severe sepsis as a result of respiratory or abdominal infections [8], and are prone to secondary infections; risk factors for these include blood transfusion [9], positive pressure respiratory support [10] and intravenous nutrition [11]. The seriousness of such infection in critically ill patients is not in doubt: more than 50% of patients with severe sepsis will die, usually from multiple organ failure [4]. It is known that a wide variety of anaesthetic and analgesic drugs negatively modulate the migration and actions of various cells in the innate immune system [5]–[7], but as these drugs act at a broad range of receptors, their cytotoxic excipients have generally been considered to be responsible [12]–[14]. Recent work has shown that Cys-loop receptors are present on immune cells, and are a potential site of drug action [15]–[17]. Here we consider whether propofol and thiopental act via GABA_A_ receptors on monocytes. These receptors are the target of a range of anaesthetics [18] and are known to modulate T-lymphocytes in mouse models of autoimmune diseases such as type 1 diabetes mellitus and experimental autoimmune encephalomyelitis [19]–[21]. Monocytes and macrophages produce and secrete GABA [21], [22], so the tonic paracrine inhibition of function seen in embryonic neurons and cerebellar granule cells may play a role in the regulation of innate immune function and inflammation. In this report we show that GABA_A_ receptors are expressed by monocytes, and that action at these proteins can explain the effects of both propofol and thiopental. The studies were performed using freshly-prepared human monocytes whenever possible, but where they were unsuitable we used the human myleomonocytic cell line (THP-1), which are routinely used as a model of human monocyte function e.g.[23].

## Results

**Figure 1:**
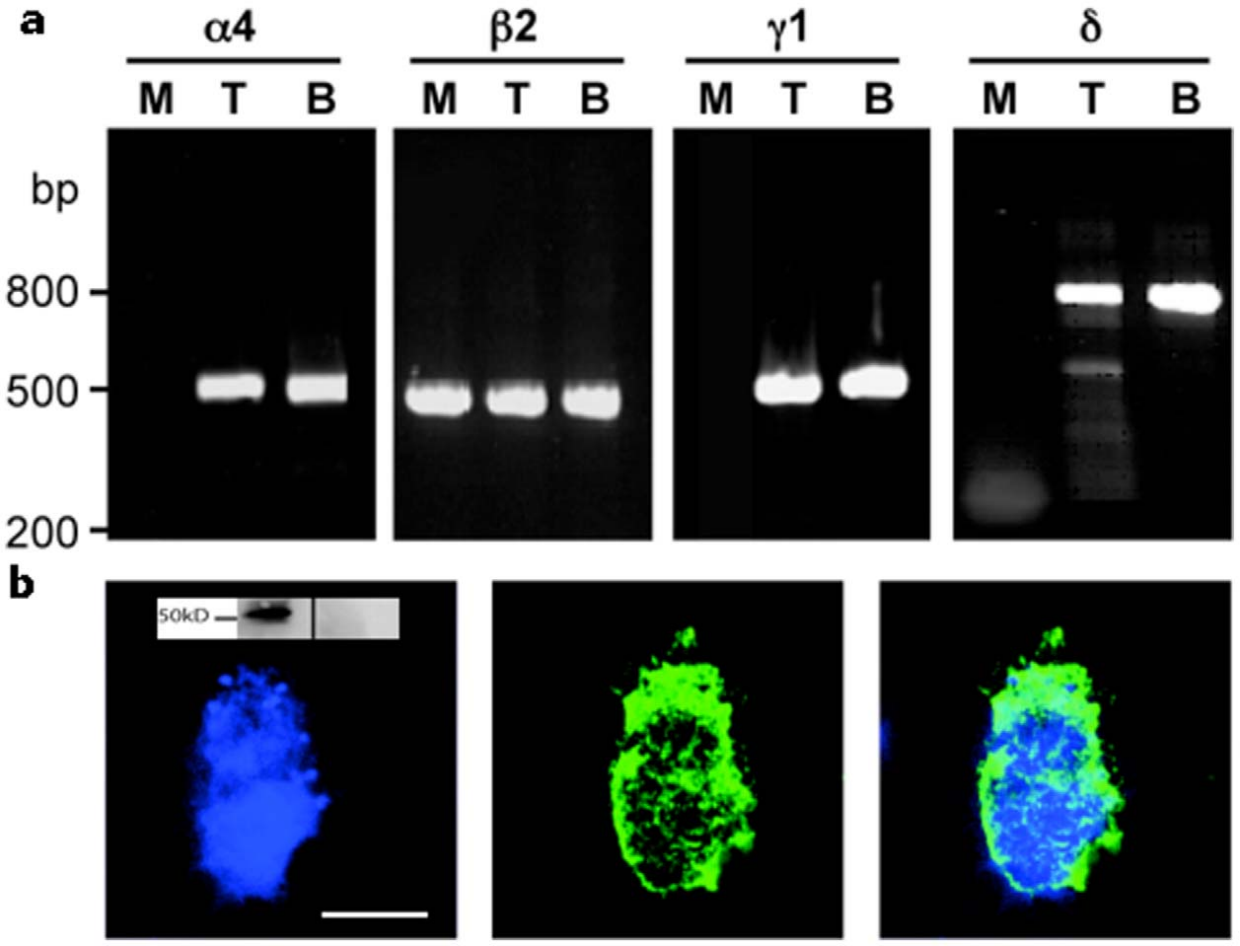
mRNA and protein expression of GABA_A_ receptor subunits in monocytic cells. **a**) Typical RT-PCR of total RNA isolated from monocyte (M) and THP-1 cell (T) lysates detecting GABA_A_ receptor subunits. Amplimers corresponding to the expected sizes were detected for α4, γ1 and δ subunits of the GABA_A_ receptor in THP-1 cells, and β2 subunits in monocytes. RNA isolated from whole human brain (B) was used as a positive control. **b**) GABA_A_ receptor β2 subunit expression in non–permeabilised human monocytes. Image of a human monocyte stained with Hoescht 33342 (left hand panel) to show the nucleus, and with a GABA_A_ receptor β2-specific polyclonal antiserum (centre) revealing cell surface β2 subunits. The right panel shows the merged image. Positive controls were human cerebral cortex and negative controls were neutrophils (data not shown). Scale bar  = 5 µm. Data are typical of at least 6 independent experiments. Inset  =  typical immunoblot of a monocyte sample (left hand side) and control (neutrophil, right hand side) probed with the β2-specific antiserum and showing expected MWt for a β2 subunit.

**Figure 2:**
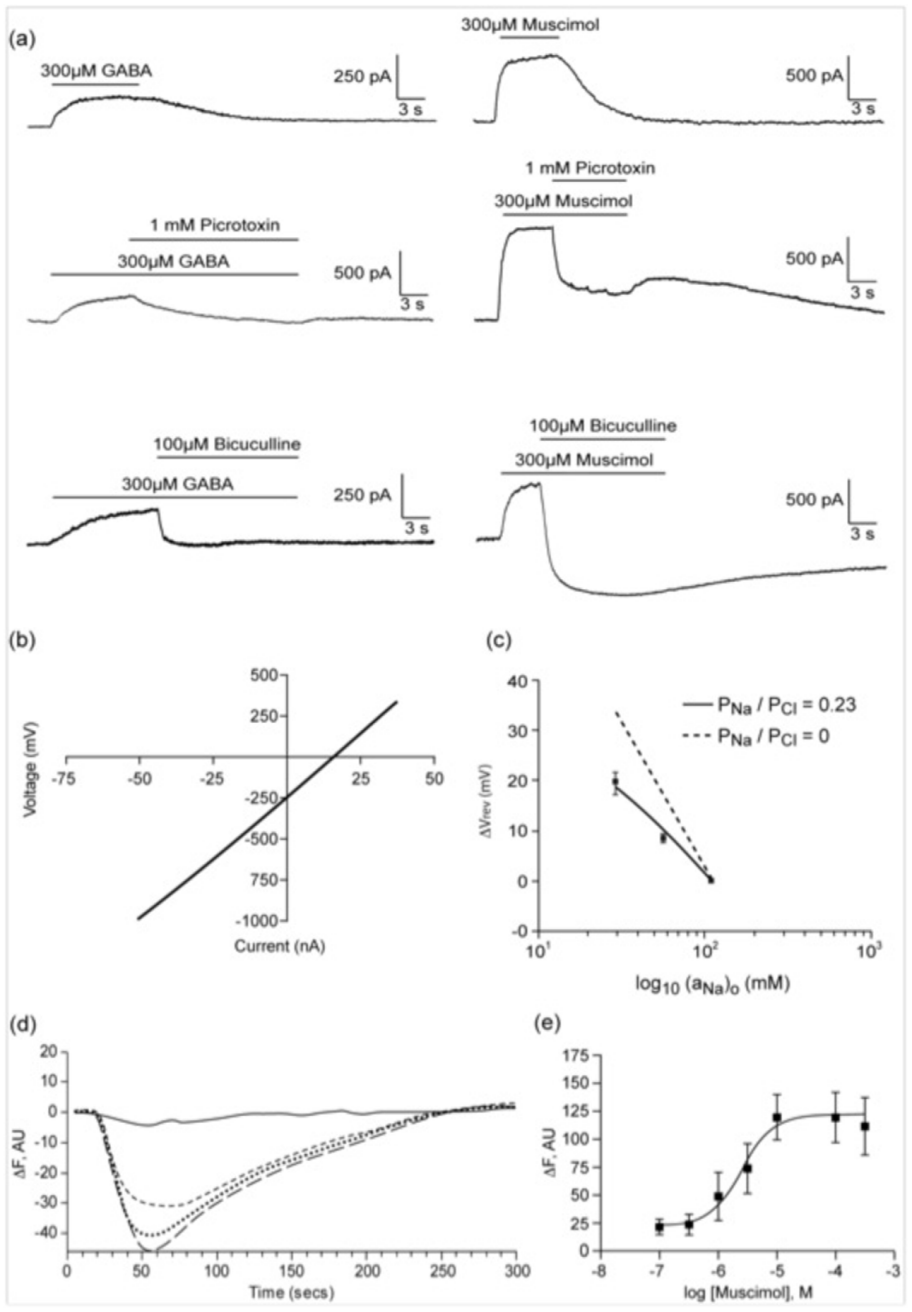
Functional responses of GABA_A_ receptors in THP-1 cells. **a**) Typical whole-cell patch-clamp traces of THP-1 cells clamped at +60 mV in the presence of GABA and muscimol; currents were blocked by the GABA_A_ receptor antagonists bicuculline and picrotoxin. **b**) Application of muscimol (300 µM) to THP-1 cells revealed a reversal potential (E_rev_) of 15.1 mV, and **c**) chloride-selectivity (P_Na_/P_Cl_) of 0.23 (n = 5). **d**) Typical Flexstation responses of THP-1 cells to different concentrations of muscimol. Buffer (continuous line) or muscimol (3, 10 and 100 µM, dashed lines) were added at 20 s. **e**) Dose curve response of THP-1 cells; EC_50_  = 2.5 µM, n = 6.

RT-PCR of RNA extracted from a human myleomonocytic cell line (THP-1 cells) and freshly prepared human monocytes showed that GABA_A_ receptor subunits are expressed in these cells. In THP-1 cells, amplimers of the correct sizes and sequences were obtained for α4, β2, γ1 and δ GABA_A_ receptor subunits (figure 1a). In fresh monocytes, only β2 subunits could be isolated (table 1). Expression of the β2 subunit protein was confirmed by immunoblotting and immunohistochemistry (figure 1b). These receptors are functional: whole cell patch clamp of THP-1 cells showed that both GABA and the GABA_A_-specific agonist muscimol elicited currents that were blocked by the GABA_A_ antagonists picrotoxin and bicuculline at 1 mM and 100 µM respectively (figure 2a). The channels predominantly conducted chloride: in whole cell patch clamp experiments the reversal potential of the GABA-induced responses was 15.1±0.7 mV (all data  =  mean ± SEM, n≥5), close to the theoretical value of 16.1 mV for a chloride channel, and under bi-ionic conditions was predominantly chloride permeable (figures 2b and 2c). Due to the small amplitudes of the whole-cell responses and the fragility of the cells, it was not possible to obtain reliable concentration-response curves. However, the use of a membrane potential-sensitive dye revealed concentration-dependent decreases in fluorescence to the GABA-selective agonist muscimol (EC_50_  = 2.5 µM, pEC_50_  = 5.61±0.22, figures 2d and e), which were inhibited by the GABA_A_ receptor antagonists bicuculline and picrotoxin with IC_50_s of 135 µM (pIC_50_  = 3.87±0.29) and 29.6 µM (pIC_50_  = 4.53±0.29) respectively. We also observed no potentiation of these effects in the presence of the benzodiazepine diazepam, and no responses to bicuculline and picrotoxin up to concentrations of 1 mM (data not shown). No specific radioligand binding could be observed with [^3^H]-GABA, [^3^H]-muscimol or [^3^H]-flunitrazepam (data not shown), which was consistent with the low levels of protein expression and small currents recorded in THP-1 cells.

**Table 1:** GABA_A_ receptor subunits detected using RT-PCR in human monocytes, THP-1 cells and human cerebral cortex (positive control); n = 4–12.

Subunit	Cerebral cortex	Human monocytes	THP-1 cells
α1	+	-	-
α2	+	-	-
α3	+	-	-
α4	+	-	+
α5	+	-	-
α6	+	-	-
β1	+	-	-
β2	+	+	+
β3	+	-	-
γ1	+	-	+
γ2S	+	-	-
γ2L	+	-	-
3	+	-	-
δ	+	-	+
ε	+	-	-
π	+	-	-
θ	+	-	-

Typical immunoblots are show in figure 1.

**Figure 3:**
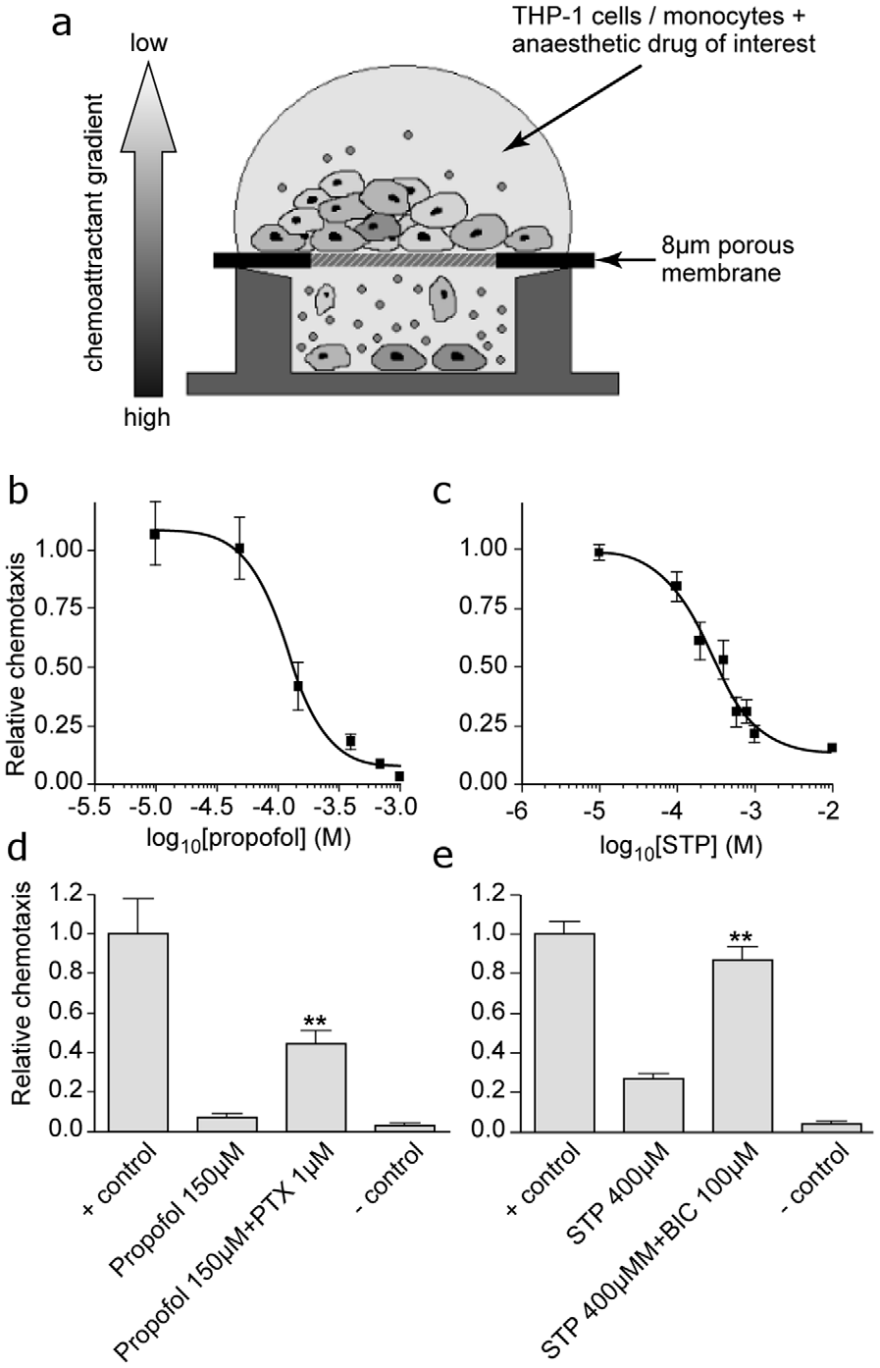
Inhibition of monocyte migration by anaesthetics is reversed by GABA_A_ antagonists. **a**) Schematic representation of the *in vitro* transwell chemotaxis apparatus used to assay human primary monocyte migration; **b and c**) Concentration-dependent inhibition of chemotaxis was observed in the presence of propofol (PPF) and sodium thiopental (STP) (IC_50_s  = 119 µM and 274 µM, respectively, n = 6); **d and e**) In the presence of either propofol (PPF) or sodium thiopental (STP), chemotaxis was significantly restored by the addition of the GABA_A_ antagonists picrotoxin (PTX) and bicuculline (BIC); (** sig diff. Mann-Whitney U test: p<0.01). Experiments were also performed with THP-1 cells and no significant differences between the behaviour of freshly-prepared human monocytes and THP-1 cells were seen (data not shown).

To examine monocyte migration we used a transwell *in vitro* migration assay (figure 3). Both THP-1 cells and fresh human monocytes migrated towards monocyte chemoattractant protein-1 (MCP-1) [24]. Propofol or thiopental inhibited this chemotaxis in a concentration-dependent fashion; maximum inhibition was 97.3±0.3% for propofol and 79.0±3.8% for thiopental, with IC_50_s of 119 µM (pIC_50_  = 3.93±0.08) and 274 µM (pIC_50_  = 3.56±0.09) respectively. To determine if this process was modulated via GABA_A_ receptors, we performed the same experiment in the presence of picrotoxin and bicuculline. Both these compounds significantly restored migration (figure 3); thiopental (400 µM) reduced migration to 26.9±2.5%, while in the presence of bicuculline (100 µM) migration was restored to 87.0±6.3%. Similarly 1 mM picrotoxin increased chemotaxis from 6.8±2.1% to 44.4±6.2% in the presence of 150 µM propofol.

**Figure 4:**
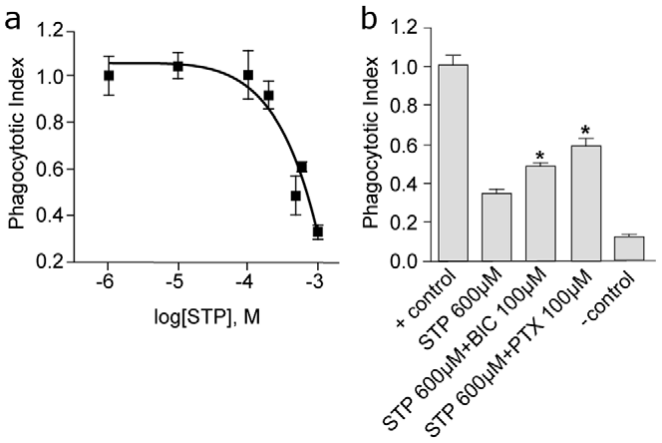
Inhibition of monocyte phagocytosis by anaesthetics is reversed by GABA_A_ antagonists. **a**) Concentration dependent inhibition of phagocytosis by thiopental in primary human monocytes; **b**) In the presence of either propofol (PPF) or sodium thiopental (STP), phagocytosis was significantly restored by the addition of the GABA_A_ receptor antagonists picrotoxin (PTX) and bicuculline (BIC) (* sig diff. Mann-Whitney U test: p<0.05).

The ability of thiopental to inhibit monocyte phagocytosis of fluorescently labelled beads was also explored to further examine monocyte function. These data (figure 4a) demonstrated that thiopental inhibits phagocytosis with an IC_50_ of 229 µM (pIC_50_  = 2.64±0.52). This inhibition was partially prevented by application of picrotoxin and bicuculline (figures 4b and 4c).

## Discussion

The data we present here show that functional GABA_A_ receptors, constructed of a combination of α1, α4, β2, γ1 and/or δ subunits, are present on monocytes. Whole cell patch clamp studies show that these receptors have properties broadly similar to CNS GABA_A_ receptors, and the anaesthetic drugs propofol and thiopental, which can also activate GABA_A_ receptors, impaired monocyte function in classic immunological chemotaxis and phagocytosis assays. The data therefore could provide an explanation as to why chronic propofol and thiopental administration can increase the risk of infection in critically ill patients.

The benzodiazepine diazepam did not affect function or modulate the effects of propofol or thiopental. Benzodiazepines have long been known to modulate GABA_A_ receptor responses [25], although more recently it was shown that this capacity critically depends on the subunit composition of the GABA_A_ receptors [26]. GABA_A_ receptors are heteromeric pentamers that most commonly contain 2α, 2β and a γ subunit, although as there are 19 GABA_A_ receptor subunits, a large number of receptor stoichiometries are possible. We detected α4, β2, γ1 and δ subunits in THP-1 cells and β2 subunits in fresh monocytes. Detecting RNA in the latter is problematic, due to high levels of RNAases and difficulties in obtaining large numbers of pure monocytes, and other subunits, such as α1, may be present. RNA for this subunit has been previously reported in monocytes which, despite lack of evidence of any other subunits, also responded to GABA_A_ receptor agonists [27]. This suggests that a likely constitution of the GABA_A_ receptors in monocytes is α4α1β2β2γ1 and/or α4α1β2β2δ, combinations typical of tonic GABA_A_ receptors responsible for slower signalling in the central nervous system [28]. GABA_A_ receptors containing α4 or δ subunits, and those lacking a γ2 subunit, would be expected to be insensitive to diazepam [29]. The detection of β2 subunits is significant, as this subunit contains a propofol-sensitive binding site, and - intriguingly - these subunits can assemble into functional receptors that respond to thiopental *in vitro* [30], [31]. Thus our RNA data are consistent with the presence of GABA_A_ receptors in monocytes that are not modulated by benzodiazepines.

This first demonstration that propofol and thiopental modulate monocyte function through actions at diazepam-insensitive GABA_A_ receptors has important clinical implications. If a patient's primary pathology is inflammatory, the immunomodulatory effects of propofol or thiopental could be therapeutic, but if it is infective they may increase the risk of sepsis. Recently the use of benzodiazepines as sedatives has been largely supplanted by propofol, but our data suggest that propofol might compromise immune cell function, while benzodiazepines would not. This raises the possibility that sedation strategies in critically ill patients could be tailored to their diagnosis, with a more traditional benzodiazepine-based approach for patients with, or at risk of, severe sepsis.

An essential question is whether our findings are relevant in terms of the concentrations of thiopental and propofol used in clinical practice. Here the concentrations at which thiopental and propofol cause a 50% reduction in monocyte chemotaxis *in vitro* are 270 µM and 120 µM respectively. High dose thiopental infusions result in a serum concentration of ∼73 mg.l^−1^, which is ∼300 µM [32], and the concentration of propofol required to prevent 50% of patients responding to a surgical stimulus equates to 38 µM [33]. There is a potential problem in that these compounds can bind to serum proteins, but this may not have large effects at low concentrations [34] and we included serum (bovine serum albumin) in our chemotaxis experiments to mimic this effect. It should also be considered that whilst half maximal effective and inhibitory (EC_50_ and IC_50_) concentrations are the conventional means of reporting experimental data, far more subtle reductions in function could have clinically relevant consequences. Thus we propose that thiopental and propofol are used in clinical practice at concentrations that have the potential to significantly diminish monocyte function *in vivo*.

An immunocompromising effect of two such widely used anaesthetics has significant consequences. Not least our data make a strong case for investigating alternative sedation strategies for patients depending on their primary pathology, and also add weight to the case for developing more precise methods for quantifying the adequacy of sedation. Clinicians titrate the doses of sedative drugs against rather blunt endpoints, tending to err on the side of caution (i.e. higher concentrations) to ensure that patients are comfortable and not distressed. Recent papers have focused on the need to minimise sedation levels in anaesthesia and critical care to reduce complications and time spent in an intensive care unit [35], [36]. While most attention has focused on the sedative effects of drugs in this situation, our data suggest that the increased potential to compromise immune function may play a part. Lower plasma concentrations or novel combinations of sedatives could significantly improve prognoses. The same applies during surgery: administering a larger anaesthetic dose than is necessary could have implications for wound healing and acquisition of postoperative infections.

Our findings also raise the intriguing possibility of administering GABA_A_ antagonists to influence immune function. Picrotoxin and securinine have been shown to positively modulate macrophage activity and enhance the clearance of bacteria [37], [38]. A highly charged non-toxic GABA_A_ antagonist that does not cross the blood brain barrier could be a means of diminishing the unwanted peripheral effects of the agonist drugs, or might even act as a novel non-sedating anti-inflammatory drug.

In conclusion we have demonstrated the clinical importance of GABA_A_ receptors on monocytes, and suggest that using anaesthetic or sedative drugs that act via different receptors could avoid immune impairment. At this stage it is not possible to quantify the extent to which GABA_A_ agonists contribute to the acquisition of infection on the intensive care unit considering the complex interplay of many pathophysiological processes in critical illness and multi-organ dysfunction. Our data suggest that the drugs used to sedate patients might be part of the problem, but also indicate possible solutions. The hypothesis that improved or different sedation strategies could reduce fatalities from septicaemia in the critically ill requires further testing, but the outcome potentially has very great clinical implications.

## Materials and Methods

### Materials

All reagents were source from Sigma Aldrich (Poole, UK) unless otherwise stated.

### Cell preparation

Peripheral blood mononuclear cells were used as the source of primary human monocytes. These had been isolated by centrifugation of Buffy coat (Blood Transfusion Service, Cambridge, UK) in Percoll gradients [39]. Monocytes were negatively isolated using immunomagnetic beads coated with monoclonal antibodies against human CD2, CD7, CD16, CD19, CD56 and CD3 as previously described (Monocyte negative isolation kit; Invitrogen, Paisley, UK) [40]. THP-1 cells (European Collection of Cell Cultures, Salisbury, Wiltshire, UK) were maintained at a density of between 4×10^5^ and 1×10^6^ cells/ml in RPMI 1640 supplemented with penicillin, streptomycin, 10% fetal calf serum and 20 µM 2-mercaptoethanol.

### RT-PCR

Total RNA was extracted using an RNAqueous-4PCR kit (Ambion, Huntingdon, UK). RT-PCR reactions were prepared using 0.2 µg of total RNA and 20 pmol oligonucleotide primers using a SuperScript One-Step RT-PCR with Platinum Taq kit (Invitrogen). Total RNA extracted from human brain was used for positive controls; reactions lacking RT were also included. The expression of the ‘housekeeping gene’ glyceraldehyde-3-phosphate dehydrogenase (GAPDH) was also examined. The primer sequences used to identify GABA_A_ subunit mRNA have been published previously [41], those for GAPDH were: ACCACAGTCCATGCCATCAC (sense) and TCCACCACCCTGTTGCTGTA (antisense). PCR conditions were: 2 min at 94°C x1; 94°C for 15 s, 55°C for 30 s and 68°C for 1 min×40; 5 min at 68°C x1. Amplimers were resolved on a 1% agarose gel, extracted and sequenced (Applied Biosystems 3130×l, Applied Biosystems, Warrington, UK). Results were compared with known mRNA sequences on the Entrez PubMed nucleotides database (http://www.ncbi.nlm.nih.gov/entrez) using ClustalW (http://www.ebi.ac.uk/Tools/clustalw2).

### Immunohistochemistry and immunoblotting

Cells were fixed with 4% paraformaldehyde in phosphate buffered saline (PBS), washed in Tris buffered saline (TBS), blocked with 3% BSA in TBS containing 0.2% Tween 20 (TTBS) and incubated overnight in a humidified chamber at 4°C with 1∶1000 β2 subunit antisera (Abcam, Cambridge, UK). After 2 washes in TTBS, the samples were incubated with FITC-conjugated secondary antibody at room temperature for 3 h. Following washing, the samples were dried and mounted in Vectashield containing 4′,6-diamidino-2-phenylindole (Vector, London, UK), and examined using confocal microscopy. Immunoblotting was performed using standard techniques with an overnight incubation using a 1∶1000 dilution of the β2 subunit antisera, followed by a 1 h incubation with an HRP-conjugated secondary antibody. Bands were detected using enhanced luminol chemiluminescent reagent (Perkin Elmer, Beaconsfield, UK).

### Whole cell patch clamp

THP-1 cells were grown on fibronectin-coated sterile cover slips. Cells were clamped at +60 mV in whole-cell configuration. Extracellular saline consisted of (mM) 140 NaCl, 5.4 KCl, 1 MgCl_2_, 1.0 CaCl_2_, 10.0 HEPES (pH 7.2). Pipettes were filled with (mM) 140 CsCl, 1.0 MgCl_2_, 1.0 CaCl_2_, 10.0 EGTA, 10.0 HEPES (pH 7.2). Currents were filtered at a frequency of 1 kHz (−3 dB) with a 4-pole low-pass Bessel filter and acquired at a sampling frequency of 110 Hz. Current-voltage relationships were studied using a voltage-ramp protocol from −80 mV to +40 mV over 1 s. Series resistance was usually less than 5.0 MΩ and voltage errors never exceeded 5 mV.

### FlexStation analysis of potentiometric dyes

THP-1 cells were washed in flex buffer (in mM 115.0 NaCl, 1.0 KCl, 1.0 CaCl_2_, 1.0 MgCl_2_, 1.0 glucose, 10.0 HEPES pH 7.4) and 1×10^5^ cells added to each well of a 96 well plate. This was centrifuged at 1000 rpm for 2 min to embed an even layer of cells, and then incubated at room temperature for 45 min in 100 µl buffer containing membrane potential dye (Blue Kit, Molecular Devices, Wokingham, UK) before being assayed in the FlexStation (Molecular Devices). Fluorescence was measured every 2 s for 300 s. At 20 s, 50 µl of muscimol was added to each well. Data was analysed using Prism (GraphPad, San Diego, USA).

### Transwell filter migration assay

MCP-1 (Peprotech, London, UK) was diluted in Gey's Balanced Salt Solution (GBSS) with 1% BSA to a final concentration of 12.5 ng.ml^−1^; 29.2 µl was added to each well of 96 well disposable chemotaxis chamber (Neuroprobe, Gaithersburg, USA) and an 8 µm polycarbonate filter membrane fitted. 1×10^5^ monocytes in 25 µl GBSS/BSA were then placed on the top of each well. The assembled chamber was incubated at 37°C in a humidified atmosphere of air and 5% CO_2_ for 90 min. Compounds under investigation were added in equal concentrations to both the top and bottom compartments of the migration chamber. After incubation, the cells were gently removed from the top of the filter with a pipette, 20 µl of ice-cold 20 mM EDTA in PBS was added to the top of each well and incubated at 4°C for 15 min. Cells that had migrated into the lower compartment of the chamber were incubated at 37°C for 60 min with 3 ml of the vital dye 3-(4,5-dimethylthiazol-2-yl)-2,5-diphenyl tetrazolium bromide (MTT; 5 mg/ml in RPMI-1640). The liquid was then carefully aspirated from the wells leaving the stained cells, and the converted formazan blue dye was solubilised using 20 µl DMSO per well. Absorbance of the converted dye was measured at a wavelength of 595 nm using an ELISA plate reader (Molecular Devices). The number of cells migrated in each well was determined by interpolation of an 8-point standard curve using Softmax Pro (Molecular Devices).

### Monocyte Phagocytosis Assay

THP-1 cells were differentiated in 200 nM phorbol 12-myristate 13-acetate (PMA) for 24 h. Fluorescent (FITC) labelled microspheres (Molecular Probes) were coated with 1% BSA (2.25×10^9^ microspheres.ml^−1^) and added to the cells in 500 µl HEPES-buffered saline (mM: 140.0 NaCl, 5.0 KCl, 2.0 CaCl_2_, 1.0 MgCl_2_, 10.0 HEPES, pH 7.4) with the compounds under investigation and incubated for 1 h. The positive control conditions were PMA alone; the negative control was unstimulated THP-1 cells. The cells were then washed with ice-cold PBS solution, fixed with 0.5% glutaraldehyde and analysed by flow cytometry using FlowJo software (version 7.5.5, Tree Star Inc., OR, USA). A phagocytosis index was calculated from the average number of beads taken up per cell.
